# Quantitative microvascular analysis in different stages of retinitis pigmentosa using optical coherence tomography angiography

**DOI:** 10.1038/s41598-024-55070-0

**Published:** 2024-02-26

**Authors:** Richul Oh, Kunho Bae, Chang Ki Yoon, Un Chul Park, Kyu Hyung Park, Eun Kyoung Lee

**Affiliations:** Department of Ophthalmology, Seoul National University College of Medicine, Seoul National University Hospital, #101, Daehak-ro, Jongno-gu, Seoul, 03080 Republic of Korea

**Keywords:** Foveal avascular zone, Inherited retinal disorders, Optical coherence tomography angiography, Retinitis pigmentosa, Vessel density, Pathogenesis, Retinal diseases

## Abstract

As retinitis pigmentosa (RP) is chronic and progressive, the chronological sequence of microvascular changes is important for understanding its pathophysiology. We aimed to investigate retinal and choroidal microvascular changes according to the RP stages. The stages of RP were classified into three stages according to the integrity and width of the inner segment ellipsoid zone: early, ≥ 2500 μm; moderate, < 2500 μm; advanced, absence. Using optical coherence tomography angiography, quantitative microvascular parameters were analyzed. In total, 91 eyes from 49 patients were included. For the superficial capillary plexus (SCP) and deep capillary plexus (DCP), perfusion densities (PDs) in the early stage (SCP: 37.32 ± 8.11%; DCP: 21.19 ± 9.15%) were greater than those in moderate (SCP: 34.16 ± 6.65%, *P* = 0.011; DCP: 15.67 ± 8.85%, *P* = 0.031) and advanced stages (SCP: 33.71 ± 9.02%, *P* = 0.030; DCP: 12.83 ± 6.29%, *P* < 0.001). The choroidal vascularity index in the early stage (0.58 ± 0.03) was greater than those in the moderate (0.57 ± 0.02, *P* = 0.017) and advanced stage (0.56 ± 0.02, *P* = 0.033). The area and perimeter of foveal avascular zone (FAZ) in advanced stage (0.44 ± 0.26 mm^2^, 2.96 ± 0.86 mm, respectively) were larger than those in early (0.26 ± 0.11 mm^2^, *P* = 0.020; 2.19 ± 0.53 mm, *P* = 0.006, respectively) and moderate stage (0.28 ± 0.13 mm^2^, *P* = 0.043; 2.24 ± 0.67 mm, *P* = 0.013, respectively). During RP disease progression, retinal and choroidal microvascular vessel density decreases in the early stage, followed by FAZ enlargement in the advanced stage.

## Introduction

Retinitis pigmentosa (RP) is a clinically and genetically heterogeneous group of inherited retinal disorders characterized by diffuse progressive dysfunction of predominantly rod photoreceptors with subsequent degeneration of cone photoreceptors and the retinal pigment epithelium (RPE)^1,2^. More than 1.5 million individuals are affected globally, with a prevalence of approximately 1:4000^3^. The visual impairment typically involves night vision and mid-peripheral vision, with gradual deterioration of central visual acuity^1^.

However, the pathogenesis of RP is not fully understood. In addition to bone spicule pigmentation on fundoscopic examination, retinal vessel attenuation is one of the most classical findings in RP^4–6^. Previous histopathological research revealed that thickening of the blood vessel walls and occlusion of their lumina correlate with sclerosis and atrophy of the retinal vasculature, which is shown as attenuated retinal arteries^7,8^. The attenuated retinal vessels are thought to reflect decreased metabolic demand of the degenerated retina, where the loss of oxygen consumption leads to increased local oxygen levels in the inner retina, which in turn results in vasoconstriction^9^.

Recent advances in optical coherence tomography angiography (OCTA) have enabled quantitative analysis of the retinal and choroidal microvasculature. Decreased retinal and choroidal vessel densities in RP have been described in previous studies^7,10–12^. Moreover, the foveal avascular zone (FAZ) area in RP was greater than that in normal eyes^11,13^. Reductions in retinal blood flow have been established using OCTA; however, questions remain regarding the relationship between vascular dysfunction and the underlying pathogenesis of RP. Whether the microvasculature is more severely impaired in more advanced RP remains to be explored. As RP is chronic and progressive, the chronological sequence of microvascular changes is important for understanding its pathophysiology. In this study, we aimed to investigate the temporal relationship of microvascular changes in RP by analyzing quantitative microvascular parameters of the retina and choroid according to the RP stage.

## Results

The medical records of 68 patients (136 eyes) diagnosed with RP were reviewed. Among the 136 eyes, 34 eyes with cystoid macular edema, four eyes with choroidal neovascularization, six eyes with epiretinal membrane, and one eye with macular hole were excluded from the analysis because comorbid retinal pathologies can affect the retinal and choroidal microvasculature.

In total, 91 eyes of 49 patients were included in the analysis. The mean age at the acquisition of OCTA was 43.9 ± 16.4 years (range, 13–78 years). Twenty-three patients were male and 26 were female. Based on the integrity and width of the preserved inner segment ellipsoid zone (ISE), 40 eyes were classified as early, 31 as moderate, and 20 as advanced groups. Table 1 shows the demographic and clinical characteristics of the included eyes according to the RP subgroup.

**Table 1:** Demographic and clinical characteristics according to the stage of retinitis pigmentosa.

	All subjects (n = 91)	Early (n = 40)	Moderate (n = 31)	Advanced (n = 20)	*P*
Age, years	43.9 ± 16.4	45.3 ± 15.7	37.6 ± 14.5	50.8 ± 18.3	0.194
Laterality, R/L	45/46	21/19	14/17	10/10	0.827
Sex, M/F	23/26	10/11	8/9	5/6	0.993
DM, n	2	0	0	2	0.027
HTN, n	6	3	1	2	0.582
VA, LogMAR	0.38 ± 0.62	0.10 ± 0.39	0.40 ± 0.45	1.00 ± 0.81	< 0.001
IOP, mmHg	16.27 ± 2.88	16.57 ± 3.01	16.50 ± 2.83	15.22 ± 2.53	0.283

*R* Right; *L* Left; *M* Male; *F* Female; *DM* Diabetes mellitus; *HTN* Hypertension; *VA* Visual acuity; *LogMAR* Logarithm of the minimum angle of resolution; *IOP* Intraocular pressure.

Statistical analyses were performed using the Kruskal–Wallis test for a continuous variable and Chi-square test for categorical variables.

Continuous variables are reported as mean values ± standard deviation. All other data area numbers.

**Figure 1:**
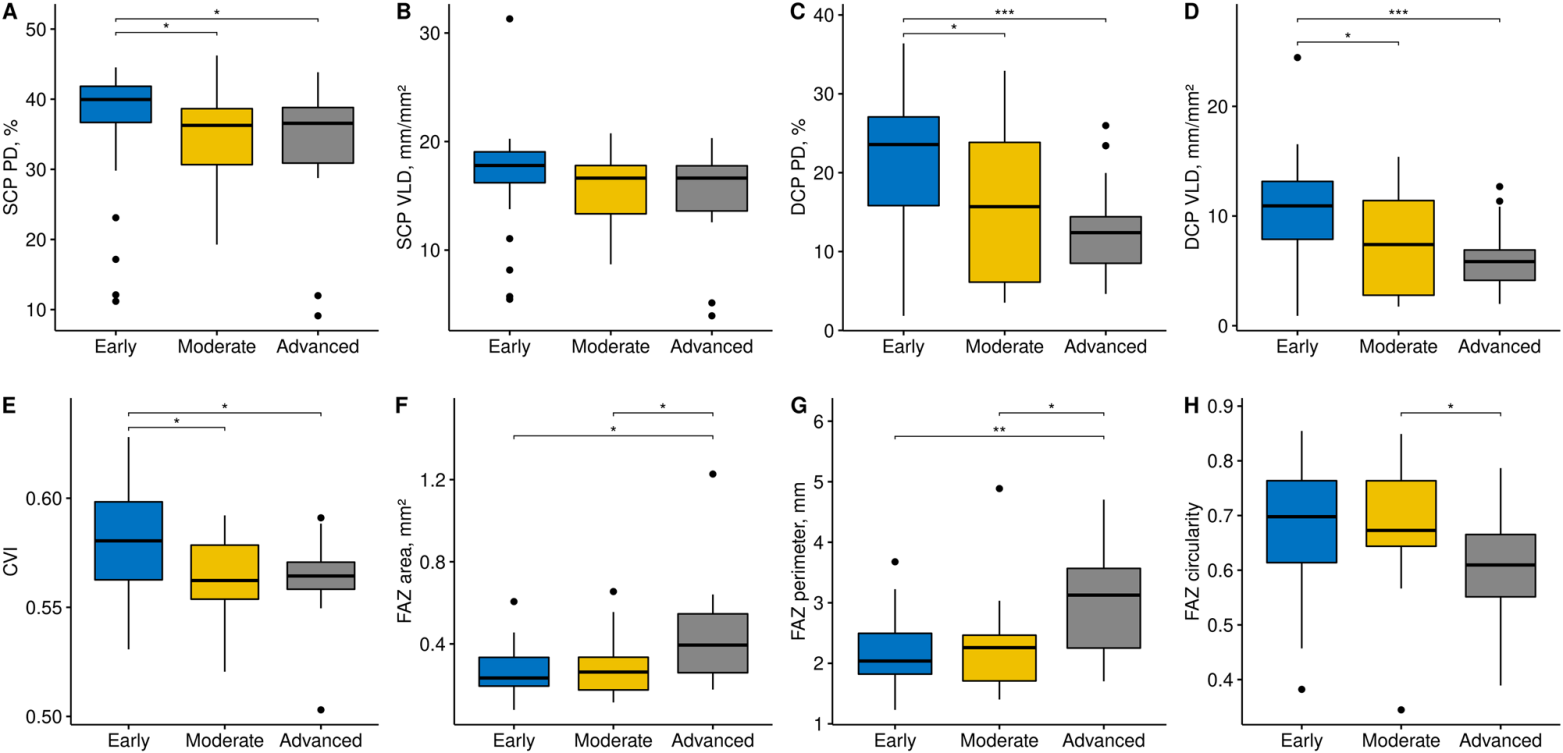
Boxplots showing parameters of optical coherence tomography angiography in different groups of retinitis pigmentosa. (**A**) Perfusion density (PD) in the superficial capillary plexus (SCP). (**B**) Vessel length density (VLD) in the SCP. (**C**) PD in the deep capillary plexus (DCP). (**D**) VLD in the DCP. (**E**) Choroidal vascularity index (CVI). The area (**F**), perimeter (**G**), and circularity (**H**) of the foveal avascular zone (FAZ). *P* values are indicated by asterisks; *P < 0.05, **P < 0.01, ***P < 0.001.

The vascular metrics for the retina and choroid are summarized in Table 2 and Fig. 1. For the superficial capillary plexus (SCP), perfusion density (PD) in the early group (37.32 ± 8.11%) was greater than that in the moderate (34.16 ± 6.65%, *P* = 0.011) and advanced groups (33.71 ± 9.02%, *P* = 0.030). For the deep capillary plexus (DCP), PD in the early group (21.19 ± 9.15%) was also greater than that in the moderate (15.67 ± 8.85%, *P* = 0.031) and advanced groups (12.83 ± 6.29%, *P* < 0.001). There was no significant difference in PD between the moderate and advanced groups for either the SCP or DCP. The vessel length density (VLD) in the DCP was greater in the early group (10.41 ± 4.82) compared with the moderate (7.53 ± 4.23, *P* = 0.030) and advanced groups (6.14 ± 3.05, *P* < 0.001). However, the VLD in the SCP was not significantly different among the three subgroups. The choroidal vascularity index (CVI) in the early group (0.58 ± 0.03) was greater than that in the moderate (0.57 ± 0.02, *P* = 0.017) and advanced groups (0.56 ± 0.02, *P* = 0.033).

**Table 2:** Retinal and choroidal microvascular features in different stages of retinitis pigmentosa.

Variable		Early (n = 40)	Moderate (n = 31)	Advanced (n = 20)	*P*	Early versus moderate	Early versus advanced	Moderate versus advanced
Perfusion density, %	Superficial	37.32 ± 8.11	34.16 ± 6.65	33.71 ± 9.02	**0.006**	**0.011**	**0.030**	0.782
	Deep	21.19 ± 9.15	15.67 ± 8.85	12.83 ± 6.29	**0.001**	**0.031**	** < 0.001**	0.278
Vessel length density, mm/mm^2^	Superficial	17.12 ± 4.17	15.63 ± 3.15	15.39 ± 4.27	**0.045**	0.066	0.161	0.752
	Deep	10.41 ± 4.82	7.53 ± 4.23	6.14 ± 3.05	**0.001**	**0.030**	** < 0.001**	0.201
Choroid vascularity index		0.58 ± 0.03	0.57 ± 0.02	0.56 ± 0.02	**0.008**	**0.017**	**0.033**	0.838
Foveal avascular zone	Perimeter, mm	2.19 ± 0.53	2.24 ± 0.67	2.96 ± 0.86	**0.007**	0.937	**0.006**	**0.013**
	Circularity	0.68 ± 0.12	0.69 ± 0.10	0.61 ± 0.10	**0.028**	0.960	0.056	**0.020**
	Area, mm^2^	0.26 ± 0.11	0.28 ± 0.13	0.44 ± 0.26	**0.022**	0.926	**0.020**	**0.043**

Statistical analysis was performed using the Kruskal–Wallis test and Mann–Whitney–U test with Hochberg correction for pairwise comparison.

Significant values with *P* < 0.05 are in bold.

**Figure 2:**
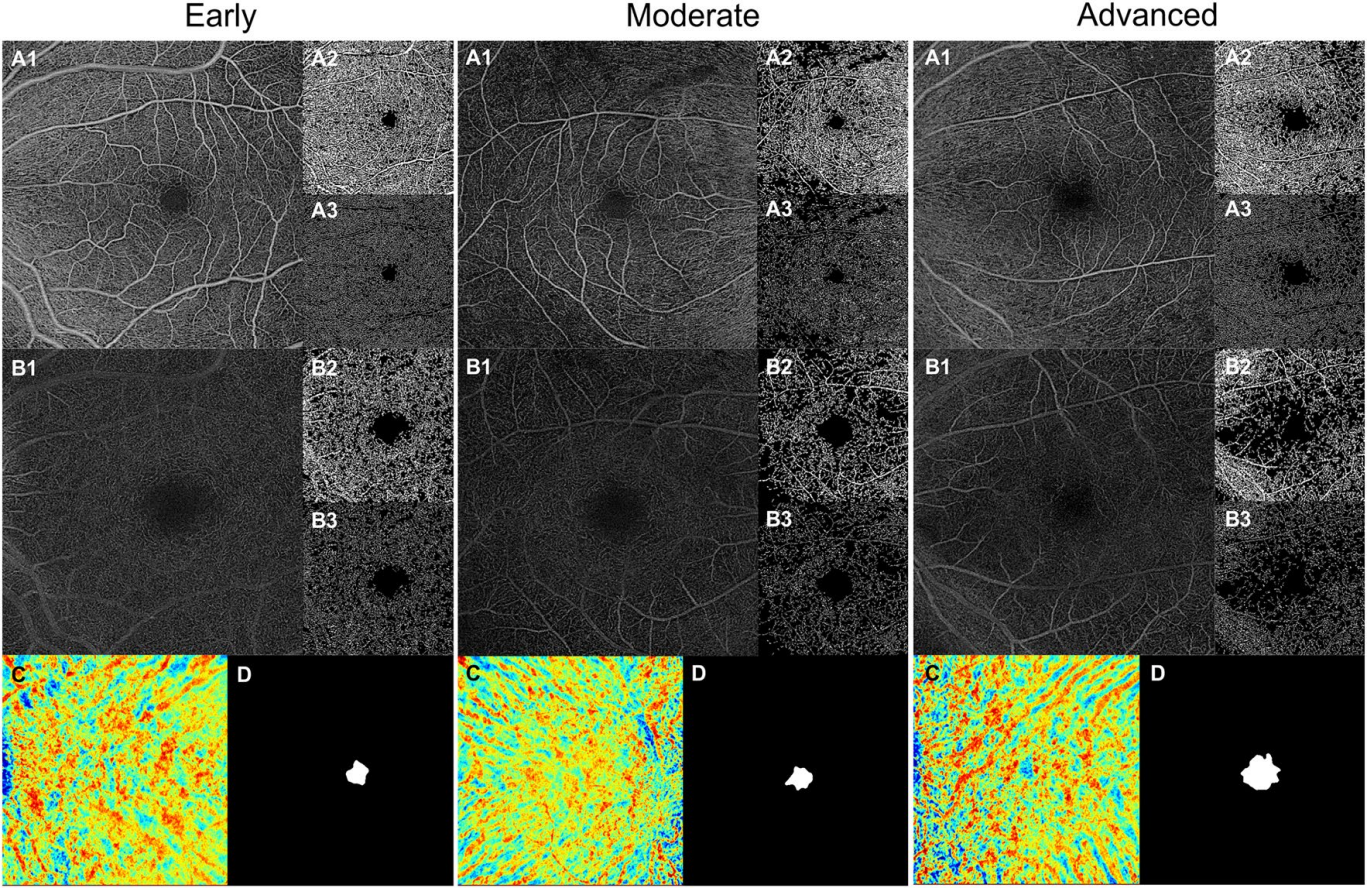
Representative examples in optical coherence tomography angiography analysis of different groups of retinitis pigmentosa. For each case, slab (**A1**), perfusion trace (**A2**), and vessel trace (**A3**) image of the superficial capillary plexus. Slab (**B1**), perfusion trace (**B2**), and vessel trace (**B3**) image of the deep capillary plexus. (**C**) Choroidal vascularity index map. (**D**) Outline and mask of foveal avascular zone.

The FAZ perimeter in the advanced group (2.96 ± 0.86 mm) was larger than that in the early (2.19 ± 0.53 mm, *P* = 0.006) and moderate groups (2.24 ± 0.67 mm, *P* = 0.013). The FAZ circularity index was decreased in the advanced group (0.61 ± 0.10) compared with the moderate group (0.69 ± 0.10, *P* = 0.020). Moreover, the FAZ area in the advanced group (0.44 ± 0.26 mm^2^) was larger than that in the early (0.26 ± 0.11 mm^2^, *P* = 0.020) and moderate groups (0.28 ± 0.13 mm^2^, *P* = 0.043). There was no significant difference in FAZ metrics between the early and moderate groups. Figure 2 shows representative cases of retinal and choroidal microvascular analyses in the three different subgroups of RP.

## Discussion

In the present study, we investigated the retinal and choroidal microvascular changes according to the different stages of RP. The PD, VLD, and CVI in the early group were greater than those in the moderate and advanced groups, whereas there was no significant difference between the moderate and advanced groups. The FAZ area in the advanced group was greater than that in the early and moderate groups, whereas there was no significant difference in the FAZ area between the early and moderate groups. These results suggest that the decrease in retinal and choroidal microvascular vessel density occurs at a relatively early stage following the enlargement of the FAZ in more advanced stages. To the best of our knowledge, this is the largest RP cohort study to investigate differences in quantitative microvascular alterations according to the severity of RP.

Several previous studies have reported retinal microvascular changes in RP. Previous studies have shown that both SCP and DCP PDs are significantly decreased in early- to late-stage RP compared with healthy eyes^10–12^. Our study is in line with previous studies and provides additional insights into the vascular pathophysiology of RP. In the early group, the PDs in the SCP and DCP were greater than those in the moderate and advanced groups. This suggests that as the disease progresses, vascular compromise in terms of PD progresses, and these changes predominantly occur in the relatively early stages of the disease. There were no differences in this parameter between the moderate and advanced groups, implying that vascular degeneration plateaued before the advanced stage of RP. The VLD showed no significant difference between groups in the pairwise comparison in the SCP, whereas the VLD in the DCP showed similar trends to the PD in the DCP. This result is consistent with Falfoul et al.^14^; they compared RP eyes with healthy eyes, and reported that these vascular alterations might begin at the level of the DCP, followed by the change in the SCP in the evolution of the disease^14^. Earlier vascular compromise of the DCP than the SCP can be explained by the reduced oxygen demand due to the death of photoreceptors. The reduced oxygen demand in the outer retina may result in an excessive amount of oxygen reaching the inner retina, leading to hyperoxia and reflex vasoconstriction of the SCP, as suggested by Eysteinsson et al.^15^.

The choroidal vascular system is crucial for oxygen and nutrition supply. In our study, the CVI in the early group was greater than that in the moderate and advanced groups. However, the CVI showed no significant difference between the moderate and advanced groups. These results imply that a decrease in the CVI might be involved in the relatively early stages of RP. Shen et al. have shown that CVI is decreased in RP eyes when compared with healthy eyes^16^; however, previous studies regarding choriocapillaris plexus (CCP) vessel density in RP were controversial. When compared with healthy eyes, one study showed reduced blood flow at the choriocapillaris layer^11^, while others showed no significant differences^10,17^. The CVI reflects middle and large choroidal vascularity, and the choriocapillaris might undergo different pathophysiology. In a study by Toto et al.^11^, the CCP was defined as a 30 μm-thick layer from the RPE, and the CCP vessel density was defined as the percentage area occupied by vessels in a 3 × 3-mm-square region of interest. This discrepancy could be attributed to the difference in the metrics used to evaluate choroidal blood flow between the previous and current studies.

The FAZ area and perimeter were greater in the advanced group than in the early and moderate groups. Moreover, the FAZ circularity index in the advanced group was lower than that in the early and moderate groups. These findings suggest that as the disease progresses, the FAZ may become enlarged and irregularly shaped. We hypothesized that the FAZ enlargement might be the secondary change followed by the degeneration of the vascular plexus. The spatial difference in the rate of vascular plexus degeneration might affect the irregularity of the FAZ. However, the study showed that the FAZ maintained its circular shape in the early and moderate stages compared to the advanced stage. As the fovea is the most important for maintaining the visual acuity, the parafoveal vascular supply should be maintained until the disease progresses, and this homeostasis might be the possible mechanism for the late change of FAZ area and perimeter. Jauregui et al.^18^ revealed that the rate of FAZ enlargement in RP eyes is greater than that in healthy eyes, reflecting that FAZ enlargement is one of the main pathogenesis. Our study provides additional information regarding the FAZ perimeter and circularity. Moreover, our study suggests that alteration of the FAZ might predominantly occur in the advanced stage, although longitudinal research is required to reveal the rate of FAZ enlargement according to the stage of RP. The utilization of these OCTA parameters can be applied clinically for new classification of RP patients, better understanding of RP pathogenesis, early detection of the disease and biomarker of disease progression, and later as a marker to evaluate improvement after new encouraging treatment such as gene therapy.

Previous studies have reported correlations between functional parameters and OCTA variables in patients with RP. Toto et al.^11^ reported a significant correlation between multifocal electroretinogram values and vessel density of the SCP and DCP. Sugaraha et al.^19^ revealed that parafoveal flow density in the deep layer and superficial FAZ area were associated with visual acuities. Moreover, Koyanagi et al.^12^ showed that parafoveal flow density in the SCP and DCP correlated with central visual parameters, visual acuities, mean deviation of the visual field test, and macular sensitivity. These studies suggested that the microvasculature was more impaired in eyes with more severe retinal degeneration; that is, more advanced RP. However, they did not reveal a chronological correlation between OCTA parameters. The strength of the present study is that it demonstrates the temporal evolution of vascular pathophysiological changes in a large number of patients with RP.

**Figure 3:**
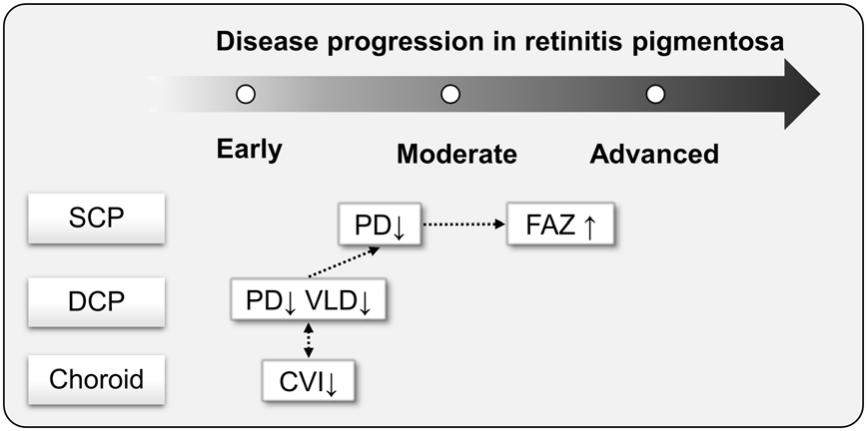
Hypotheses regarding temporal evolution of retinal and choroidal vascular changes in retinitis pigmentosa. *SCP* Superficial capillary plexus; *PD* Perfusion density; *FAZ* Foveal avascular zone; *DCP* Deep capillary plexus; *VLD* Vessel length density; *CVI* Choroidal vascularity index.

Our hypothesis regarding the chronological relationship of vascular pathophysiology in RP is illustrated in Fig. 3. The results of the current study suggest that PD and VLD reduction in the DCP with CVI reduction in patients with RP may occur first in the relatively early stage, followed by decreased blood flow in the SCP, with FAZ enlargement occurring at a more advanced stage. Recently, Lu et al.^20^ proposed a new hypothesis on the pathogenesis of RP using OCTA findings. The proposed hypothesis was mainly based on case–control studies and functional correlations with structural abnormalities. Our hypothesis regarding the degeneration of the DCP and SCP of the retinal vessels is in line with the previously proposed hypothesis. However, our study provides additional evidence regarding the FAZ and CVI. Choroidal vascular alterations may occur in earlier stages, whereas FAZ changes may occur in more advanced stages.

This study had some limitations. First, the lack of longitudinal data makes it difficult to assess the causal relationship in retinal and choroidal vascular alterations. However, the strength of this study is that we quantitatively analyzed OCTA vascular metrics in different stages of RP, rather than simply comparing RP eyes to healthy controls. Future longitudinal studies with longer follow-up periods using OCTA will provide a better understanding of the pathophysiology of RP. Secondly, the sample size was relatively small. Despite the small sample size, this is the largest study to our knowledge, and the differences in vascular parameters between the stage groups were statistically significant, which makes our results sufficiently reliable for further studies. Third, both eyes of the patients were included unless they had other significant macular pathologies that could alter the microvasculature. Both eyes may have similar vascular characteristics, as RP is a genetically determined degenerative disease that usually involves bilateral eyes. However, bilateral involvement is not always symmetrical. Although both eyes of the same patient were in the same stage groups, these eyes did not have the same structural and functional abnormalities, which implies that the microvasculature might be different. The inclusion of both eyes of one patient in the analysis may have affected the results of this study. Fourth, as only 24.5% of the patients in the current study were genetically identified, analysis of hemodynamic changes according to the causative gene was not performed, and the genetic heterogeneity of the study participants may have influenced the results of the study. Further studies, including genetic analyses, may provide a better understanding of RP pathophysiology. Fifth, the classification of the disease stages based on the width of the ISE was reported previously but not yet validated methodology. Future study utilizing a more validated stage system with additional functional parameters are needed to confirm our results.

In conclusion, PD, VLD, and CVI were greater in the early RP group than in the moderate and advanced RP groups. Vascular compromise in the DCP seems to occur earlier, and the SCP is affected later in disease evolution. The FAZ area in the advanced group was greater than those in the early and moderate groups. Therefore, in the progression of RP, a decrease in retinal and choroidal microvascular vessel density occurs in the earlier stages, followed by FAZ enlargement in the more advanced stages.

## Materials and methods

We retrospectively reviewed the medical records of consecutive patients diagnosed with RP who underwent OCTA examination at Seoul National University Hospital, Seoul, Republic of Korea. All procedures were conducted according to the tenets of the Declaration of Helsinki, and the study design was approved by the Institutional Review Board of Seoul National University Hospital (IRB 2105-086-1219), which waived the need for written informed consent because of the retrospective design of the study and the use of deidentified patient information. The exclusion criteria were as follows: (1) eyes with macular pathology (epiretinal membrane, cystoid macular edema, etc.) other than photoreceptor loss, which can cause secondary impact on the structural integrity of the retinal and choroidal vasculature; (2) clinical presentation similar to RP, such as a history of trauma or uveitis, hydroxychloroquine retinopathy, or autoimmune retinopathy; and (3) OCTA images with a signal strength index < 8.

**Figure 4:**
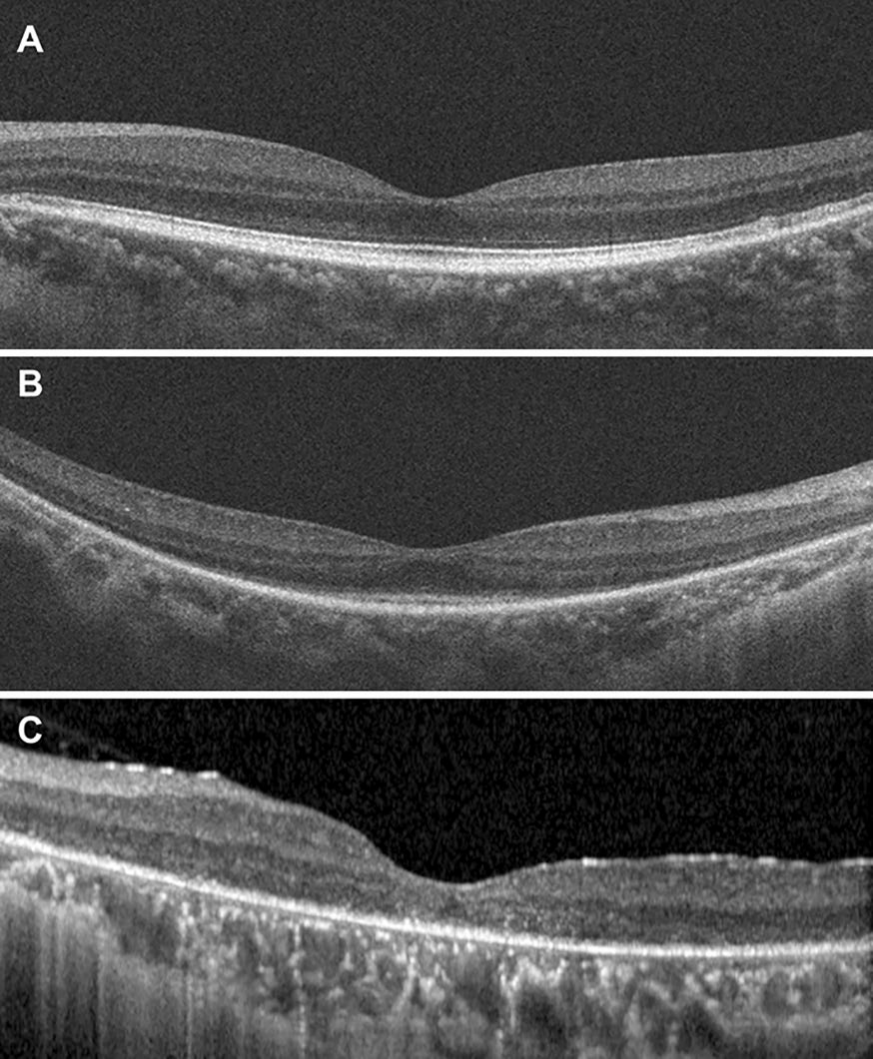
Representative examples of different groups of retinitis pigmentosa (RP) based on the width of the preserved inner segment ellipsoid zone (ISE). (**A**) Early group exhibiting the preserved ISE of more than 2500 μm from the fovea. (**B**) Moderate group demonstrating the preserved ISE of less than 2500 μm from the fovea. (**C**) Advanced group with absence of visible ISE.

The diagnosis of RP was based on clinical history including night blindness and decreased visual acuities, characteristic fundus appearance including bone spicule pigmentation, corresponding visual field defect, and electroretinography findings^2,21,22^. Eyes were classified into three groups based on RP stage according to the integrity and width of the preserved ISE on spectral-domain optical coherence tomography (SD-OCT) (Heidelberg Spectralis; Heidelberg Engineering, Heidelberg, Germany) taken on the same day of OCTA^23^. The early group exhibited a preserved ISE of more than 2500 μm from the fovea, whereas the moderate group showed constricted but preserved ISE within 2500 μm from the fovea on OCT scans. The advanced and moderate groups were distinguished based on the visibility of the preserved ISE on OCT images within 2500 μm from the fovea; the preserved ISE was visible in the moderate group, but not in the advanced group (Fig. 4)^23^.

All patients were imaged using a 100-kHz swept-source OCTA instrument (PLEX Elite 9000; Carl Zeiss Meditec, Dublin, CA) that uses a laser with a central wavelength of 1060 nm with a bandwidth of 100 nm. Angiographic 6 × 6 mm^2^ scans centered on the fovea were acquired by trained photographers. Images with a signal strength index < 8 were excluded.

All images were analyzed using the ARI Network (Zeiss Portal V.Portal version 6.0–0005, https://arinetworkhub.com, accessed 15 Feb 2023), a cloud-based automatic image processing and analysis website. The images were manually evaluated to confirm proper placement-centered fovea. The retinal layer and choroid segmentations were reviewed and manual adjustments were performed. The PD and VLD of the SCP and DCP were analyzed. For the DCP, projections from the SCP were removed using an automatic algorithm in the ARI Network. The PD was defined as the total area of perfused vasculature per unit area in a region of measurement. The VLD was defined as the total length of the perfused vasculature per unit area in a region of measurement. It was measured in units of inverse millimeters. The PD and VLD were measured in fovea-centered 6-mm circles. The CVI was defined as the ratio of the choroidal luminal area to the total choroidal area, measured in a fovea-centered 5-mm circle. The FAZ was assessed in terms of the SCP and FAZ perimeter, circularity, and area were analyzed. Automatic segmentations of the FAZ were manually reviewed and incorrectly segmented FAZ images were excluded from the further analysis. All manual evaluations and adjustments were performed by two independent examiners (R.O. and E.K.L). The examiners were masked for the stage of RP.

All statistical analyses were performed using R software (version 4.2.1.; R Foundation, Vienna, Austria). Shapiro’s test was performed to investigate the normality of the parameters. The Kruskal–Wallis test and Mann–Whitney U test with Hochberg correction for pairwise multiple comparison were performed to compare continuous variables between the groups. The chi-square test was used for categorical variables. Continuous variables are presented as mean ± standard deviation. The level of statistical significance was set at *P* < 0.05.

### Ethical approval

All procedures performed in studies involving human participants were in accordance with the ethical standards of the Seoul National University Hospital (IRB 2105-086-1219) and with the 1964 Helsinki declaration and its later amendments or comparable ethical standards.

### Informed consent

Informed consent was waived because of the retrospective design of the study and the use of deidentified patient information.

## Data Availability

The datasets used and/or analysed during the current study available from the corresponding author on reasonable request.

## References

[1] Ferrari S (2011). Retinitis pigmentosa: Genes and disease mechanisms. Curr. Genom..

[2] Hamel C (2006). Retinitis pigmentosa. Orphanet J. Rare Dis..

[3] Pagon RA (1988). Retinitis pigmentosa. Surv. Ophthalmol..

[4] Hartong DT, Berson EL, Dryja TP (2006). Retinitis pigmentosa. Lancet.

[5] Ma Y (2012). Quantitative analysis of retinal vessel attenuation in eyes with retinitis pigmentosa. Investig. Opthalmol. Vis. Sci..

[6] Berson EL (1993). Retinitis pigmentosa: The Friedenwald lecture. Investig. Ophthalmol. Vis. Sci..

[7] Milam AH, Li ZY, Fariss RN (1998). Histopathology of the human retina in retinitis pigmentosa. Prog. Retin. Eye Res..

[8] Li ZY, Possin DE, Milam AH (1995). Histopathology of bone spicule pigmentation in retinitis pigmentosa. Ophthalmology.

[9] Grunwald JE, Maguire AM, Dupont J (1996). Retinal hemodynamics in retinitis pigmentosa. Am. J. Ophthalmol..

[10] Parodi MB (2017). Vessel density analysis in patients with retinitis pigmentosa by means of optical coherence tomography angiography. Br. J. Ophthalmol..

[11] Toto L (2016). Macular features in retinitis pigmentosa: Correlations among ganglion cell complex thickness, capillary density, and macular function. Investig. Opthalmol. Vis. Sci..

[12] Koyanagi Y (2018). Optical coherence tomography angiography of the macular microvasculature changes in retinitis pigmentosa. Acta Ophthalmol..

[13] Inooka D (2018). Quantification of macular microvascular changes in patients with retinitis pigmentosa using optical coherence tomography angiography. Investig. Opthalmol. Vis. Sci..

[14] Falfoul Y (2020). Multimodal imaging in retinitis pigmentosa: Correlations among microvascular changes, macular function and retinal structure. J. Curr. Ophthalmol..

[15] Eysteinsson T, Hardarson SH, Bragason D, Stefánsson E (2014). Retinal vessel oxygen saturation and vessel diameter in retinitis pigmentosa. Acta Ophthalmol..

[16] Shen C (2020). Choroidal vascular changes in retinitis pigmentosa patients detected by optical coherence tomography angiography. BMC Ophthalmol..

[17] Takagi S (2018). Optical coherence tomography angiography in patients with retinitis pigmentosa who have normal visual acuity. Acta Ophthalmol..

[18] Jauregui R, Park KS, Duong JK, Mahajan VB, Tsang SH (2018). Quantitative progression of retinitis pigmentosa by optical coherence tomography angiography. Sci. Rep..

[19] Sugahara M (2017). Optical coherence tomography angiography to estimate retinal blood flow in eyes with retinitis pigmentosa. Sci. Rep..

[20] Lu B-W, Wu G-P, Xie L-K (2021). In depth understanding of retinitis pigmentosa pathogenesis through optical coherence tomography angiography analysis: A narrative review. Int. J. Ophthalmol..

[21] Fahim A (2018). Retinitis pigmentosa: Recent advances and future directions in diagnosis and management. Curr. Opin. Pediatr..

[22] Heckenlively JR, Yoser SL, Friedman LH, Oversier JJ (1988). Clinical findings and common symptoms in retinitis pigmentosa. Am. J. Ophthalmol..

[23] Yoon CK, Bae K, Yu HG (2022). Longitudinal microstructure changes of the retina and choroid in retinitis pigmentosa. Am. J. Ophthalmol..

